# High-dose proton beam therapy for sinonasal mucosal malignant melanoma

**DOI:** 10.1186/1748-717X-9-162

**Published:** 2014-07-23

**Authors:** Hiroshi Fuji, Shusuke Yoshikawa, Masako Kasami, Shigeyuki Murayama, Tetsuro Onitsuka, Hiroya Kashiwagi, Yoshio Kiyohara

**Affiliations:** 1 Divisions of Proton Therapy, Shizuoka Cancer Center Hospital, Nagaizumi, Shizuoka 411-8777, Japan; 2 Divisions of Dermatology, Shizuoka Cancer Center Hospital, Nagaizumi, Shizuoka 411-8777, Japan; 3 Divisions of Pathlogy, Shizuoka Cancer Center Hospital, Nagaizumi, Shizuoka 411-8777, Japan; 4 Divisions of Head and Neck Surgery, Shizuoka Cancer Center Hospital, Nagaizumi, Shizuoka 411-8777, Japan; 5 Divisions of Opthalomology, Shizuoka Cancer Center Hospital, Nagaizumi, Shizuoka 411-8777, Japan; 6 Divisions of Radiation Oncology, National Center for Child Health and Development, 2-10-1 Okura, Setagaya, Tokyo 157-8535, Japan

**Keywords:** Mucosal melanoma, Proton beam therapy, Radiotherapy, Sinonasal melanoma

## Abstract

**Background:**

The significance of definitive radiotherapy for sinonasal mucosal melanoma (SMM) is sill controvertial. **T**his study was to evaluate the role of high-dose proton beam therapy (PBT) in patients with SMM.

**Methods:**

The cases of 20 patients with SMM localized to the primary site who were treated by PBT between 2006 and 2012 were retrospectively analyzed. The patterns of overall survival and morbidity were assessed.

**Results:**

The median follow-up time was 35 months (range, 6–77 months). The 5-year overall and disease-free survival rates were 51% and 38%, respectively. Four patients showed local failure, 2 showed regrowth of the primary tumor, and 2 showed new sinonasal tumors beyond the primary site. The 5-year local control rate after PBT was 62%. Nodal and distant failure was seen in 7 patients. Three grade 4 late toxicities were observed in tumor-involved optic nerve.

**Conclusion:**

Our findings suggested that high-dose PBT is an effective local treatment that is less invasive than surgery but with comparable outcomes.

## Introduction

Sinonasal mucosal melanoma (SMM) is a rare disease accounting for 0.3–2% of all malignant melanomas in North America [1]. Its prognosis is much worse than that of cutaneous malignant melanoma; the overall 5-year survival rate of patients with SMM is reported to be 34% [2] whereas that of patients with head and neck cutaneous melanoma is reported to be 80% [3]. Surgery has been the main option for eradicating the disease [4-6]. However, in a review of cases from a high-volume cancer center in North America, recurrence of the primary disease was observed in 50% of cases [7-9].

For patients with SMM, radiotherapy has been employed for palliation or as adjuvant treatment for surgery. However, a nationwide survey recently performed in the United States revealed that radiotherapy has a limited role in improving overall patient survival [3]. Definitive radiotherapy for SMM has been applied selectively because of tumor radioresistance and the propensity for metastases to distant organs. Furthermore, in the case of SMM, intensive radiotherapy is only applicable to a small proportion of patients because the tumors are frequently surrounded by radiosensitive critical tissues, e.g., the optic nerve, cranial nerves, and the brain stem.

Highly conformal beam delivery techniques are promising measures with which to deliver definitive high-dose radiotherapy in patients with SMM. Intensity-modulated radiotherapy and particle beam therapy have both been reported to facilitate the delivery of high doses to the residual tumor while minimizing exposure to the surrounding normal tissues [10-14].

In a short-term analysis, the primary disease control rate associated with high-dose conformal proton beam therapy was reported to be promising [14]. These data propose a definitive role for proton beam therapy; however, the actual efficacy of this treatment with escalating prescribed dose needs to be established for long-term tumor control and the morbidity in critical organs at the irradiated sites needs to be evaluated. Furthermore, the significance of the local treatment for improved overall survival and the pattern of relapse needs to be analyzed with a sufficient follow-up time. The purpose of this study therefore was to describe the long-term course of patients undergoing definitive high-dose PBT for localized SMM.

## Patients and methods

### Patients

This retrospective analysis was approved by the internal review board of our institution. From 2006 to 2010, 20 consecutive SMM patients underwent definitive PBT at our institution. The feasibility of surgical resection was discussed among proficient head and neck surgeons and proposed to the patients. Eight patients were inoperable and others refused surgery because of irreversible impairment of organs at the skull base by surgery. Written informed consent was obtained from patients. All patients had biopsy-proven mucosal malignant melanomas. Histological diagnoses were based on the findings of hematoxylin and eosin staining, with confirmation using immunostaining in some cases. An experienced pathologist classified SMM cases according to the state of cellular differentiation. Differentiated cells were epithelioid, localized to interconnected nests, and/or spindle-shaped in a fascicular pattern. Undifferentiated cells were smaller and characterized by an incohesive architecture. Disease extent was evaluated using computed tomography (CT) and magnetic resonance imaging (MRI) scans. Of 20 patients, 18 underwent fluorodeoxyglucose (FDG)-positron emission tomography (PET) for the detection of regional and distant lesions. Melanomas of the upper aerodigestive tract were classified in accordance with the criteria listed in the seventh edition of the TNM Staging System of the Union Internationale Contre le Cancer for melanoma of the upper aerodigestive tract. Ten of 20 tumors involved the unilateral optic nerve. Table 1 presents the clinical characteristics of patients.

**Table 1:** Patient characteristics

**Category**	**Number**
Gender	
Male:Female	12:8
Median age (years, range)	74 (55–81)
Tumor size (mm, range)	19.5 (7.0–130.2)
Primary site	
Nasal cavity	12
Paranasal sinus	8
Clinical stage	
T3N0M0	9
T4aN0M0	8
T4bN0M0	3
Pathological subtype	
Small cell type	11
Spindle cell type	5
Epithelioid type	4

The median time interval between diagnosis and the start of PBT was 4.8 months (range, 0.5–56.2 months). Nine patients had recurrent disease after local surgery before being referred to our institute. Seven patients underwent 1–8 cycles of systemic chemotherapy before PBT. All patients underwent definitive PBT because gross tumor volume was observed at the start of PBT. Sixteen patients underwent concurrent or adjuvant multi-agent chemotherapy consisting of dacarbazine, nimustine, and vincristine or dacarbazine, nimustine, cisplatin, and tamoxifen. The treatment period of concurrent or adjuvant chemotherapy was 8 months (4–12 months).

### Proton beam therapy

The gross tumor volume (GTV) was defined as the gross extent of the tumor as observed on CT or MRI images. Areas showing elevated FDG-PET signals were also included in the GTV. The clinical target volume (CTV) was defined as GTV with a 2–10 mm margin. Areas with mucosal alteration and inflammatory tissue as seen on a T2-weighted image were also included in the CTV. The planning target volume (PTV) was defined by adding a 3-mm margin to the GTV and CTV, referred to as the PTV_(GTV)_ and PTV_(CTV)_, respectively.

A proton beam has a unique depth-dose curve, the so-called Bragg peak and deposits a maximum energy at a designated depth. With this physical feature, the proton beam provides highly conformal dose deposition for SMM. The proton beam used in this study was 150 MV, passive scattering beam. The biological effectiveness of proton on using PBT differs from that of photons on using conventional radiation because of this unique mechanism of energy deposition. On considering the relative biological effect of a proton beam, the dose was reported in grays relative biological effectiveness, Gy (RBE), which was equivalent to the physical dose in Gy multiplied by 1.1. A hypofractionated treatment schedule of 3.5 Gy (RBE) per fraction administered daily for a total dose of 70 Gy (RBE) in 20 fractions was applied.

**Figure 1:**
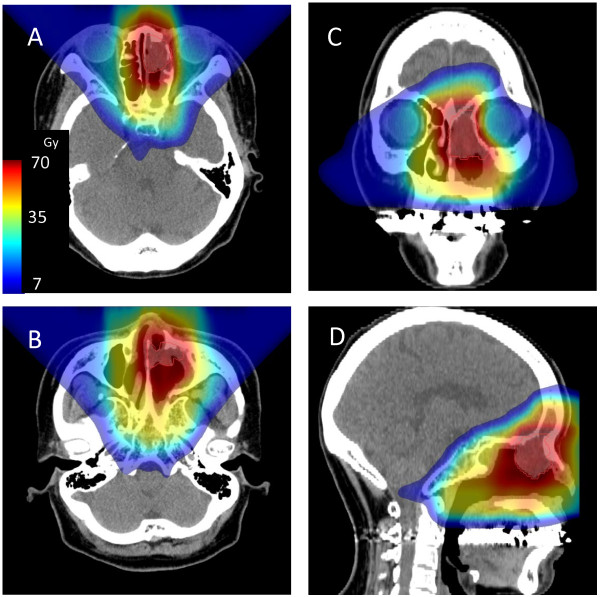
**A representative treatment plan of proton beam therapy for sinonasal mucosal melanoma.** Dose distribution on trans-axial **(A, B)**, coronal **(C)**, and sagittal **(D)** images with color scale. Colors depict the high-dose area on the gross tumor and the mid-dose area on the clinical target volume.

Treatment planning was performed such that 90% of the PTV_(GTV)_ received 95% of the prescribed dose and PTV_(GTV)_ received more than 40 Gy. Simultaneous integral boost technique was used to deliver different dose to two target volumes, PTV_(GTV)_ and PTV_(CTV)_ in single fraction. In each treatment time, PTV_(GTV)_ irradiated 3.5 Gy and PTV_(CTV)_ irradiated more than 2 Gy by using two or more field.A constraint dose of 50 Gy (RBE) was set for the optic nerve and chiasma except in cases of a tumor involving the optic nerve or chiasma (Figure 1). Dose reduction was applied for the brain stem if the dose exceeded 54 Gy (RBE). The head and neck were immobilized using thermoplastic shells during treatment sessions. Orthogonal fluoroscopy was used before every treatment session to verify beam localization. A passive scattering proton beam was used. The patterns of irradiated tumor response were assessed using MRI, CT, and PET imaging, performed every 3–6 months. Acute toxicities were scored according to the National Cancer Institute (USA) Common Toxicity Criteria version 3.0. Late toxicities were evaluated according to Radiation Therapy Oncology Group and European Organization for Research and Treatment of Cancer late radiation morbidity scoring schemes.

### Statistical analyses

Overall survival, disease-free survival, and local control rates were estimated using the Kaplan–Meier method. All events were calculated from the first day of PBT to the last day of follow-up. Local failure comprised both progression of the primary tumor and appearance of a new lesion at the sinonasal site after PBT. Standard errors for estimated survival were compared using a log-rank test for association among potential prognostic factors. A *p* value of ≤0.05 was considered statistically significant. All statistical analyses were performed using PASW 17.0 (PASW, Chicago, Illinois).

## Results

### Tumor recurrence and survival

**Figure 2:**
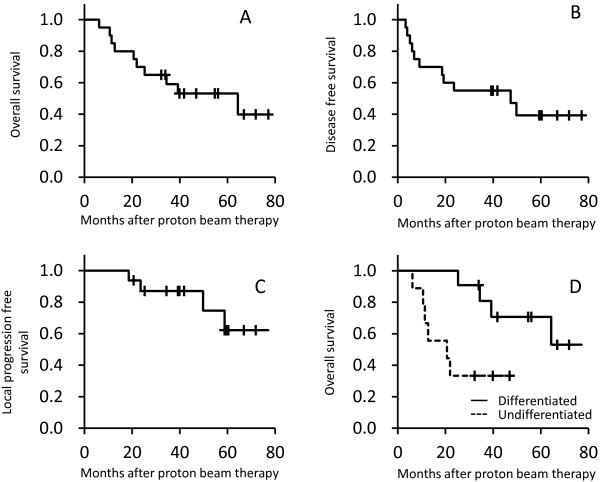
**Overall survival (A), disease-free survival (B), and local disease-free (C) rates of patients who underwent proton beam therapy for sinonasal mucosal melanoma.** The overall survival curves of patients with differentiated morphology were compared with those of patients with undifferentiated morphology and other morphologies (dotted line), and cellular morphology was found to be a significant prognostic factor **(D)**.

The median follow-up period was 35 months (range, 6–77 months). For all patients, the 3-year and 5-year overall survival rates were 68% and 54%, respectively, with a median survival time of 39 months (Figure 2A). The 3-year and 5-year progression-free survival rates were 60% and 52%, respectively (Figure 2B). At the time of analysis, 10 patients were alive. Eight patients had died due to disease progression. Four patients showed local failure at sinonasal site: 2 patients presented with primary tumor progression at 47 and 50 months after PBT and 2 presented with recurrence of melanoma in the sinonasal region beyond the GTV at 19 and 24 months after PBT. For all patients, the 3-year and 5-year actuarial local control rates were 70% and 62%, respectively (Figure 2C).

Distant metastases were observed 7 patients (liver, 3; lung, 2; brain, 1; and intestine, 1). Three patients showed lymph node involvement after PBT.Differentiated morphology presenting as epithelioid and spindle cell type cells was associated with significantly better overall survival (p = 0.018) (Figure 2D). Other candidate prognostic factors including T stage, use of concurrent chemotherapy, FDG-PET signal level, and tumor size did not show any significant association with survival.

### Treatment toxicity

Details of acute toxicity are presented in Table 2. All patients with mucositis were scored as having grade 1–3 severity. All patients with dermatitis, except for 1, experienced grade 1–3 severity. In addition, 1 patient who underwent concurrent chemotherapy experienced grade 4 thrombocytopenia. Excluding this case, overall, the hematological toxicities were mild (of less than grade 2 severity).

**Table 2:** Acute toxicities of proton beam therapy

**Type**	**Grade**	**Number (%)**
Mucositis	1–2	14 (70)
	3	5 (25)
Dermatitis	1–2	18 (90)
	3	1 (5)
Keratitis	1–2	2 (10)
Eyelid function disorder	1–2	8 (40)
Thrombocytopenia	4	1 (5)

**Figure 3:**
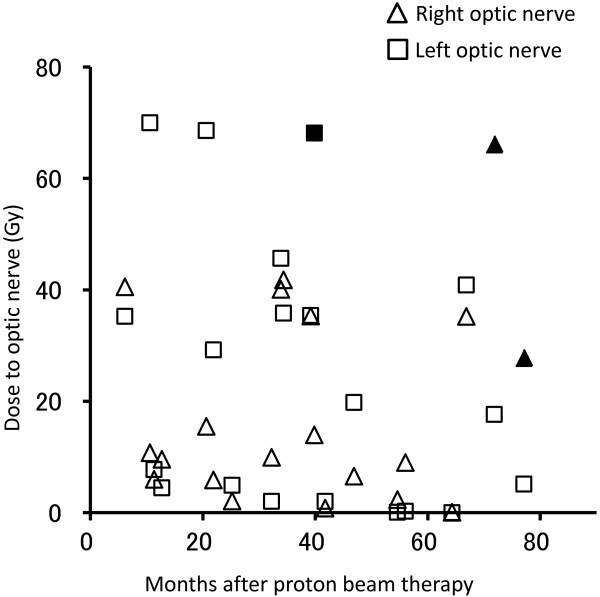
**The dose received by the optic nerve was plotted against the follow-up time to determine the relationship between irradiation dose and visual impairment onset.** The filled symbols correspond to optic nerves with visual impairment.

Late toxicities of were described in Table 3. Grade 4 toxicity observed in the cohort was optic nerve disorder. Three patients showed unilateral visual impairment after PBT. In 2 of these patients, the optic nerve was irradiated with >68 Gy and a decrease in visual function was observed 20 and 39 months after PBT. The third patient, whose right optic nerve was irradiated with <36 Gy showed right visual impairment 20 months after treatment. Images taken at the time revealed expansion of the tumor and optic nerve compression. Although tumor expansion was transient, the patient’s visual impairment did not improve. The relationship between dosing to the optic nerve and follow-up time is depicted in Figure 3.

**Table 3:** Late toxicities of proton beam therapy

**Type**	**Grade**	**Number (%)**
Nasal congestion	1	6
Epistaxis	1-2	3
Watering eyes	1-2	8
Osteonecrosis	2	5
Optic nerve disorder	4	3
Middle ear inflammation	2	7

Five patients presented with osteonecrosis in irradiated field. Initially, those patients suffered with the pain of affected bone. However, a course of antibiotics relieved the pain and enable to take food. Radiation-induced grade 2 otitis was observed in 7 patients; this could be controlled with antibiotic administration in all cases. Watering eyes due to lacrimal duct obstruction were most frequent late adverse events.

## Discussion

In this study, high-dose PBT resulted in favorable primary disease control. Most reviews on the management of head and neck mucosal melanoma suggest that radiotherapy has only a minor role and is mostly used as adjuvant treatment. However, it has been suggested that these treatments could provide an option for eradication of primary disease. A study of 85 carbon ion-treated cases reported a 5-year actuarial local control rate of 75% [12,13]. Short-term analysis of PBT also showed promising outcomes with a 3-year local control rate of 86% [14].

The current study showed that the primary disease was controlled in all patients within 3 years. The higher primary disease control rate most likely contributed to the improved overall survival of the cohort. The 5-year overall survival rate achieved here is comparable with those reported for surgery [2,8].

There are two cases developed recurrent disease outside of primary gross disease. Although in-transit spreading is known to be a major feature of mucosal melanoma, we have few knowledge for detecting microscopical infiltration of mucosal melanoma. Recent advantage of high sensitive imaging technology, like FDG-PET and other more specific imaging technique or both could be useful tool for defining irradiation target volume of mucosal melanoma. However, defining the border of tumor abutted brain or occulomotor is still challenging, because the both show higher uptake of FDG. Another type of radiotracer specific for melanin in melanoma cells, known as a benzamide derivative could be useful for defining the extension of tumor arose head and neck region [15].

Comparison between treatment outcomes of carbon ion beam and those of proton beam do not suggest apparent superiority of carbon ion treatment. Reported local control rate 3 years after carbon ion treatment ranged from 65% to 85% [12,13]. Current study showed that local control rate of 87% at 3 years after treatment. Better local control rate, 75% at 5 years after carbon ion treatment has been reported [13]. However, more censored cases with lower long term survival rate of the study remains the uncertainties of long term local disease control rate. Short term analysis of carbon ion beam and proton beam implemented for head and neck melanoma at same institute showed identical local control rate at 2 years after treatment [16].

Here, the analyses involved dose escalation and hypofractionated schemes as well as other definitive therapies with other particle beam therapy series. The prescribed doses of those particle beam treatments can be converted to 84–91 Gy in equivalent dose in 2 Gy fractions.(EQD2) of a standard fractionation scheme. These are 1.2–1.5 times higher than those employed for other head and neck tumors. Nevertheless, 2 cases of primary tumor relapse were observed 4 years after PBT. Delayed recurrence of irradiated tumors needs to be recognized as a possible characteristic of high-dose radiotherapy.

Reported intensive radiotherapy for mucosal melanoma is usually scheduled for 1–4 days/week [10-14,16]. In contrast, the current treatment plan involved a conventional schedule of 5 days/week. In terms of the deposited dose at the target, the 3.5-Gy (RBE) fraction size used here is regarded as hypofractionated but has been reported to be effective for eradicating mucosal melanoma. However, a treatment planning technique known as simultaneous integrated boost (SIB) facilitates a decreased dose per each fraction (i.e., 2 Gy (RBE) per fraction) for critical structures in the CTV. The use of the SIB technique here, even in a hypofractionated schedule, enabled the application of a constraint dose and an estimation of toxicity based on the knowledge of standard daily fractionated treatment.

We encountered 2 cases of optic nerve impairment after ablative dose delivery to the nerve. For these patients, the intention of treatment was both eradication of the tumor and preservation of contralateral visual function. Therefore, differentiated doses were employed for optic nerves. The affected optic nerve received 64 Gy, whereas the contralateral site received <50 Gy. Thus, the primary disease was controlled for 4-years and contralateral vision was preserved, suggesting that this treatment is an acceptable option for patients with unilateral optic nerve involvement or those offered optic nerve transection.

The establishment of prognostic factors to identify patients who would benefit from PBT is essential. In the current study, differentiated cellular morphology was a significant survival factor, However, an obvious limitation of the current study is the small sample size. A future analysis of a large cohort of patients treated with PBT would help reinforce the significance of cellular morphology as a prognostic indicator.

We did not implemented prophylactic cervical lymph node irradiation because the prophylactic treatment plays a limited role for improvement of survival. Gilligan reported 8 cases developed lymph node recurrence among 28 patients who underwent definitive radiotherapy for sinonasal melanoma [17]. Of eight patients, six patients emerged with concomitant disease of primary tumor recurrence or distant metastasis. In current study, we observed three cases developed neck lymph node metastasis among 20 cohort. Of three, two patients presented with recurrence at primary site or other organs. One patient without other recurrent disease was salvaged with neck dissection.

In the present study, chemotherapy was administered to 16 of 20 patients, which likely played a substantial therapeutic role because a gradual decrease in tumor markers was noted during treatment. Considering the diverse backgrounds of patients with respect to treatment modalities, the current analysis was not appropriate for evaluating chemotherapeutic benefits. Chemotherapy-induced cytotoxicity during and after the PBT is a concern; however, no patients receiving chemotherapy suffered from unexpected treatment interruptions and only 1 case of grade 4 hematological toxicity was encountered. Therefore, concurrent chemotherapy was feasible, but its significance for tumor control remains to be determined.

## Conclusion

Hypofractionated high-dose PBT reported here improved the local control rate associated with SMM. The sustained control of the primary lesion resulted in a favorable survival rate, comparable to that achieved by surgery in patients with certain tumor types.

## Competing interests

The authors declare that they have no competing interest.

## Authors' contributions

HF contributed to the concepts and design of the study. HF, SY, MK, SM, KH collected the data and all authors contributed to the interpretation of the data. HF drafted the article. All authors commented on the first draft, revised the manuscript and approved the final version.

## References

[B1] Thompson LD Wieneke JA Miettinen M Sinonasal tract and nasopharyngeal melanomas: a clinicopathologic study of 115 cases with a proposed staging system Am J Surg Pathol 2003 27 594 611 12717245 10.1097/00000478-200305000-00004

[B2] Freedman HM DeSanto LW Devine KD Weiland LH Malignant melanoma of the nasal cavity and paranasal sinuses Arch Otolaryngol 1973 97 322 325 4699540 10.1001/archotol.1973.00780010332008

[B3] Tseng WH Martinez SR Tumor location predicts survival in cutaneous head and neck melanoma J Surg Res 2010 167 192 198 21176922 10.1016/j.jss.2010.10.008 PMC3077472

[B4] Medina JE Ferlito A Pellitteri PK Shaha AR Khafif A Devaney KO Fisher SR O'Brien CJ Byers RM Robbins KT Pitman KT Rinaldo A Current management of mucosal melanoma of the head and neck J Surg Oncol 2003 83 116 122 12772206 10.1002/jso.10247

[B5] Day TA Hornig JD Sharma AK Brescia F Gillespie MB Lathers D Melanoma of the head and neck Curr Treat Options Oncol 2005 6 19 30 15610712 10.1007/s11864-005-0010-5

[B6] Bradley PJ Primary malignant mucosal melanoma of the head and neck Curr Opin Otolaryngol Head Neck Surg 2006 14 100 104 16552267 10.1097/01.moo.0000193176.54450.c4

[B7] Stern SJ Guillamondegui OM Mucosal melanoma of the head and neck Head Neck 1991 13 22 27 1989926 10.1002/hed.2880130104

[B8] Patel SG Prasad ML Escrig M Singh B Shaha AR Kraus DH Boyle JO Huvos AG Busam K Shah JP Primary mucosal malignant melanoma of the head and neck Head Neck 2002 24 247 257 11891956 10.1002/hed.10019

[B9] Mendenhall WM Amdur RJ Hinerman RW Werning JW Villaret DB Mendenhall NP Head and neck mucosal melanoma Am J Clin Oncol 2005 28 626 630 16317276 10.1097/01.coc.0000170805.14058.d3

[B10] Combs SE Konkel S Thilmann C Debus J Schulz-Ertner D Local high-dose radiotherapy and sparing of normal tissue using intensity-modulated radiotherapy (IMRT) for mucosal melanoma of the nasal cavity and paranasal sinuses Strahlenther Onkol 2007 183 63 68 17294109 10.1007/s00066-007-1616-2

[B11] Suit H Urie M Proton beams in radiation therapy J Natl Cancer Inst 1992 84 155 164 1311773 10.1093/jnci/84.3.155

[B12] Yanagi T Mizoe JE Hasegawa A Takagi R Bessho H Onda T Kamada T Okamoto Y Tsujii H Mucosal malignant melanoma of the head and neck treated by carbon ion radiotherapy Int J Radiat Oncol Biol Phys 2009 74 15 20 19046826 10.1016/j.ijrobp.2008.07.056

[B13] Mizoe JE Hasegawa A Jingu K Takagi R Bessyo H Morikawa T Tonoki M Tsuji H Kamada T Tsujii H Okamoto Y Organizing Committee for the Working Group for Head Neck Cancer Results of carbon ion radiotherapy for head and neck cancer Radiother Oncol 2012 103 1 32 37 22321201 10.1016/j.radonc.2011.12.013

[B14] Zenda S Kawashima M Nishio T Kohno R Nihei K Onozawa M Arahira S Ogino T Proton beam therapy as a nonsurgical approach to mucosal melanoma of the head and neck: a pilot study Int J Radiat Oncol Biol Phys 2011 81 135 139 20950948 10.1016/j.ijrobp.2010.04.071

[B15] Cachin F Miot-Noirault E Gillet B Isnardi V Labeille B Payoux P Meyer N Cammilleri S Gaudy C Razzouk-Cadet M Lacour JP Granel-Brocard F Tychyj C Benbouzid F Grange JD Baulieu F Kelly A Merlin C Mestas D Gachon F Chezal JM Degoul F D'Incan M (123)I-BZA2 as a melanin-targeted radiotracer for the identification of melanoma metastases: results and perspectives of a multicenter phase III clinical trial J Nucl Med 2014 55 15 22 24263087 10.2967/jnumed.113.123554

[B16] Demizu Y Fujii O Terashima K Mima M Hashimoto N Niwa Y Akagi T Daimon T Murakami M Fuwa N Particle therapy for mucosal melanoma of the head and neck Strahlenther Onkol 2014 190 186 191 24362502 10.1007/s00066-013-0489-9

[B17] Gilligan D Slevin NJ Radical radiotherapy for 28 cases of mucosal melanoma in the nasal cavity and sinues Br J Radiol 1991 64 1147 1150 1773274 10.1259/0007-1285-64-768-1147

